# Microbiology and ecology are vitally important to premedical curricula

**DOI:** 10.1093/emph/eov014

**Published:** 2015-07-20

**Authors:** Val H. Smith, Rebecca J. Rubinstein, Serry Park, Libusha Kelly, Vanja Klepac-Ceraj

**Affiliations:** ^1^Department of Ecology and Evolutionary Biology, University of Kansas, Lawrence, KS, USA,; ^2^Department of Biological Sciences, Wellesley College, Wellesley, MA, USA,; ^3^Department of Systems and Computational Biology and; ^4^Department of Microbiology and Immunology, Albert Einstein College of Medicine, Bronx, NY, USA

**Keywords:** premedical curricula, microbiology, human microbiome, ecology

## Abstract

Despite the impact of the human microbiome on health, an appreciation of microbial ecology is yet to be translated into mainstream medical training and practice. The human microbiota plays a role in the development of the immune system, in the development and function of the brain, in digestion, and in host defense, and we anticipate that many more functions are yet to be discovered. We argue here that without formal exposure to microbiology and ecology—fields that explore the networks, interactions and dynamics between members of populations of microbes—vitally important links between the human microbiome and health will be overlooked. This educational shortfall has significant downstream effects on patient care and biomedical research, and we provide examples from current research highlighting the influence of the microbiome on human health. We conclude that formally incorporating microbiology and ecology into the premedical curricula is invaluable to the training of future health professionals and critical to the development of novel therapeutics and treatment practices.

## INTRODUCTION

The influential American microbiologist D. H. Bergey was an advocate for adding bacteriology as an essential component of the general training of biology students and those seeking to obtain medical training as early as 1915 [1]. One hundred years later, in 2015, it is clear that many organ systems and physiological functions in the human body are modulated by small molecules derived from the microbiota and that the microbiome is a key determinant of human health (Table 1). Moreover, the human body can be viewed as a complex and multifaceted ecosystem, and human health can be interpreted in part as a product of the ecosystem services that are delivered by its resident microbiota [2]. As stressed by Zhou *et al.* [3], each of us is composed of diverse habitats that are exposed to and subsequently can respond to variations in our external environment. In addition, the dynamics of microbial communities associated with these habitats can be strongly modulated by local interactions with our immune, endocrine and nervous systems [3]. Each habitat provides a unique niche space for the growth and survival of indigenous and invading microbes [3], and shifts of the human body from a ‘healthy’ non-diseased state to a diseased condition are often accompanied by major alterations in microbial growth and community composition (Table 1). We thus argue here that formal inclusion of microbiology and ecology is essential in the premedical biology curriculum because of the intricate and interwoven relationships that we share with our microbial partners: archaea, bacteria, viruses and microeukaryotes such as fungi.

**Table 1.** Examples of medical specialties and organ systems where the microbiome has been suggested to play a role in health and disease

Medical specialties	Organ systems	Function	Role of microbiome	Conditions associated with altered communities	Refs
Andrology/ Gynecology/ Obstetrics	Reproductive	Production of sex hormones, production of gametes, milk production, support of embryo/fetus until birth	Modification and deconjucation of steroid hormones in the gut, defense against pathogens, microbiota transfer to fetus	Possibly a role in male infertility, bacterial vaginosis, antibiotics lead to lower estrogen levels	[4-10]
Cardiology, Hematology	Cardiovascular	Nutrient delivery, temperature modulation	The gut microbiota contributes to the synthesis of trimethylamines generated from choline and carnitine, which are further oxidized to trimethylamineoxide (TMAO) in the liver. TMAO is correlated with cardiovascular events	Cardiovascular disease	[11-13]
Dermatology	Integumentary	Protection against pathogens	Protective immunity: skin microbiota interacts with the immune cells in the skin; antimicrobial peptide production and colonization resistance	Atopic dermatitis, psoriasis	[14-19]
Endocrinology, Psychiatry, Neuroscience, Neurology, Ophthalmology, Otolaryngology	Endocrine, Brain and Nervous	Hormone secretion, detection, processing and regulation of many body processes	Regulation of host hormones, gut-brain axis- modulation of behavior, mood	Eating disorders, neurodegenerative and neurodevelopmental disorders	[20-24]
Gastroenterology	Digestive	Processing and digestion of food, waste removal	Defense against pathogens, digestion, synthesis of vitamins, breakdown of food components and xenobiotics	Mouth: periodontitis; gut: diabetes, ulcerative colitis, Crohn disease	[25-28]
Immunology, Oncology	Lymphatic/Immune	Returns fluid to blood, defense against pathogens	Immune system development and training, mucosal immunity	Inflammatory bowel disorders, allergies, autoimmune diseases	[29-33]
Nephrology, Urology	Urinary	Waste removal; removal of excess fluid	Unknown; in the past considered sterile	Gut microbiota link to renal stone formation; bladder cancer	[34]
Orthopedics	Musculo-skeletal	Body support and movement, temperature homeostasis	Bone mass regulation either via the immune system, hormones or microbial metabolites	Infections due to mislocalization of microorganisms	[35]
Pulmonology	Respiratory	O_2_/CO_2_ gas exchange	Defense against pathogens, mucosal immunity	Chronic obstructive pulmonary disorder (COPD), asthma	[36-38]

In this review, we highlight the role of ecological and microbe–host interactions in human health, and we outline how exposure to microbial ecology in premedical curricula can affect clinical practice. The broader topic of how the human ecosystem interacts with environmental ecosystems, although equally important, is not covered in this review as it has been discussed elsewhere [39–41]. We also provide brief examples that demonstrate the important potential role of the human gut microbiome under four different patient settings: (i) the administration of antibiotics, (ii) the presence of metabolic disorders, (iii) the development of cancer and (iv) the pharmacokinetics of drugs within the patient. For each of these four examples, we highlight the potential integration of practices in the clinic with discoveries in basic microbiome research and ecology.

## WHY INTEGRATE MICROBIOLOGY AND ECOLOGY INTO THE PREMEDICAL CURRICULA?

Efforts to characterize microbial communities residing within the human body during the last decade have greatly increased our understanding of microbial community composition and diversity in health and disease. However, despite characterized connections between the human microbiota and health, a profound disconnect currently exists between research on the human microbiome and the applied health fields. This disconnect is reflected in the limited exposure to the principles of microbiology and ecology that medical professionals receive during their premedical and medical education and training. Here, when we refer to premedical training, we are referring to undergraduate education. Admission requirements to medical schools do not include microbiology and ecology, and the required biology coursework is mostly centered around genetics and cell biology with an emphasis on human biology. In medical school, microbiology is often taught in the context of pathogenesis. Moreover, the principles of ecology, which broadly apply to the human microbiome [2], are not yet well integrated into standard premedical courses.

Perhaps because formal classroom exposure to the principles of microbiology and ecology is often absent from premedical curricula (Table 2), many human health professionals approach disease primarily through the lens of mammalian anatomy and physiology. This educational framework leads to a lack of integration of clinical practices with basic microbiological and ecological research.

**Table 2.** Current minimum background needed for MCAT 2015 and acceptance to Medical School in the United States [42]

Medical School Prerequisites/MCAT 2015 Preparation	Number of required semesters of coursework
Biology	2
Biochemistry	1
General Chemistry	2
Organic Chemistry	2
Physics	2
Psychology	1
Sociology	1

**Figure 1.**
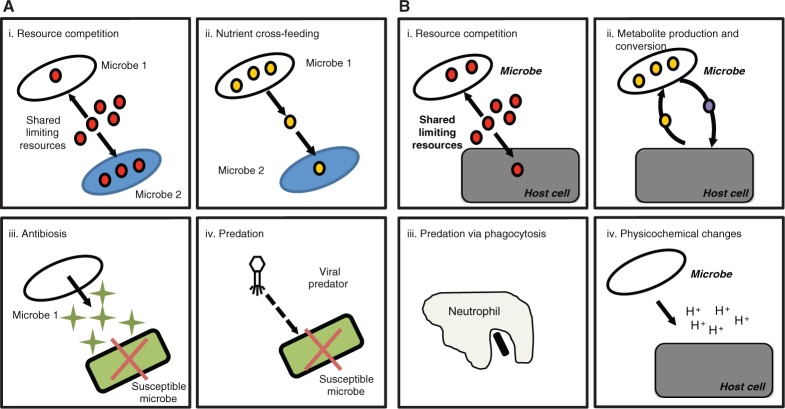
Examples of internal and external factors that can lead to conditions associated with altered microbial communities (modified from Fig. 4 in [58]). **A.** *Key microbe–microbe interactions*. Four important kinds of ecological interactions can strongly regulate the growth and population dynamics of a microbial community residing in or upon a human host. (i) *Resource competition*. The ability of multiple microbial species to compete for growth-limiting resources such as the essential nutrient glucose (red circles) may in part determine their local survival and population dynamics. Note that microbes can also compete for space and for docking sites. (ii) *Nutrient cross-feeding*. Microbial species 1 produces an essential nutrient such as folate (orange circles) that is in turn consumed by and enables the persistence of a co-resident auxotrophic species (Microbe 2) that requires this resource for growth. (iii) *Antibiosis*. One microbial species or strain (Microbe 1) may produce an antibiotic (yellow triangles) that is excreted and inhibits or kills another susceptible microorganism (Microbe 2). (iv) *Predation*. Attacks by a predator (in this example, a bacteriophage virus) results in the infection and death of susceptible prey (in this case, a gut bacterium). (Inspired by and greatly revised from Figure 1 by Seth and Taga [59]). **B.** *Key microbe–host interactions*. Four important kinds of ecological interactions can strongly influence the growth and population dynamics of interacting microbial and host cells. (i) Resource competition. Members of the host’s resident microbiome may compete with host cells for growth-limiting resources such as the essential nutrient glucose (red circles). (ii) *Metabolite production, conversion and nutrient cross-feeding*. One cell type (in this case, the host cell) may produce an essential resource (orange circles) which is then consumed and metabolized to a new metabolite by microbial cells; this microbial metabolite (purple circles) is excreted and is subsequently consumed and used by host cells. Microorganisms can generate metabolites and bacterial components, either of which can interact with receptors on host cells, or have other pharmacological effects on multiple host pathways. (iii) *Predation*. Predation of microbial cells (black rectangle) may occur by phagocytic host cells. (iv) *Physicochemical changes*. Microbial cells may excrete non-nutrient metabolic products (in this case, protons) that can alter the local environment and influence the growth and reproduction of host cells. (Inspired by and greatly revised from Figure 1 by Seth and Taga [59]).

Several examples of the diverse ecological principles that apply to the human host and its associated microbiome are outlined in Box 1, and examples of the kinds of microbe–microbe and microbe–host interactions that can occur within the human body are shown in Fig. 1. The integration of ecology with (i) the biochemistry of microbial metabolic processes and (ii) the interactions of the microbiome with human physiology, e.g. immune responses, is lacking in the more topic-segregated courses taught currently. Teaching these concepts concurrently will provide a common language to encourage communication and the exchange of ideas among medical students, clinicians and basic researchers. We also believe that there is great value in combining reductionist approaches (e.g. a focus on the molecular biology and biochemistry of the toxin A and B proteins which lead to *Clostridium difficile* colitis) with broader systems-based approaches (e.g. a focus on ecological responses of the gut microbial community during *C. difficile* infection) to better understand the human microbiome’s contributions to health and disease.

Box 1. Key ecological topics and principles that apply to the human host and its associated microbiome

*Population Ecology*. A population is a group of individuals of the same organism or cell type that co-occur in the same location and time. For example, Costerton *et al.* [43] stress that most microbial pathogens must persist and multiply in their infected system in order to cause deleterious effects. Thus, whether the initial infection event involves a single virion, fungal propagule or bacterial cell, the invader typically must replicate to large population densities before its presence significantly influences the health of its human host. Similarly, the replication and growth of immune cells (e.g. protective B and T lymphocytes), as well as potential pathogen targets (e.g. uninfected red blood cells within the bloodstream that can be targeted by the *Plasmodium* malaria parasite), are cellular populations [44]. Moreover, Smith [45] discusses relationships for resource-limited growth of populations of a hypothetical pathogen (P) and of hypothetical host cells (H), and Smith et al. [46] have recently demonstrated extremely strong effects of pre-infection T cell population growth rate on virus replication following experimental infection with a hybrid HIV-SHIV retrovirus.

*Metapopulation Ecology.* Populations must also be considered in a spatial context. Multiple populations of the same organism or cell type can co-occur in the same time, but at different locations: thus, a metapopulation is a ‘population of populations’ [47]. These spatially dispersed subpopulations occur in patches of suitable habitat surrounded by areas of unsuitable or as-yet uninvaded habitat. Individual subpopulations are connected by the spatial movements of migrating individuals, and thus any given subpopulation within the entire metapopulation can be reestablished and rescued from extinction by colonization events from distant subpopulations [47]. Mittelbach [47] provides important examples of metapopulation dynamics such as source–sink interactions that we consider to be potentially relevant to human host–human microbiome interactions.

*Community Ecology*. A community is defined as a group of different organisms, species, or cell types that co-occur in the same location and time. Community ecologists thus grapple with and attempt to predict the structure and dynamics of multispecies ensembles that live within the same habitat, landscape or region [48]. For example, the human circulatory system contains a complex community of differentiated blood cell types that have different cell architectures and functions. Similarly, the human gut contains trillions of coexisting and often closely interacting microbes of different phylogenetic origins. Every human can be viewed as a unique set of microbial assemblages occupying different habitats across the human body governed by the fundamental processes of community ecology [2]. We know relatively little about how within-host microbial communities respond to local and regional factors that affect key processes such as host reproduction, resistance to novel microbes or microbial transmission rates [49]. However, among the multiple community modules outlined by Holt and Dobson 48], we suggest that at least six kinds of interactions potentially may apply to humans and their microbiome: *food chain* (a linearly arrayed, hierarchical network of consumers and their resources); *exploitative competition*, also known as resource competition (in which two or more species compete directly for a growth-limiting nutrients); *interference competition*, also known as contest competition (in which individuals interfere with the resource acquisition and survival of other individuals, e.g. via the production of toxins); *niche partitioning* (in which two or more species reduce the magnitude of their interspecific competition by specializing upon different resources); *predation on competing prey* (in which the outcome of resource competition between two prey species is influenced by the presence of a shared predator); *apparent competition* (the appearance of resource competition between two non-interacting prey species that are differentially consumed by a shared predator); and *keystone predation* (in which a shared predator facilitates the coexistence of prey species). Robinson *et al.* [50] have recently reviewed the ecology of host-associated microbial communities, and Fierer *et al.* [51] have applied concepts developed by plant and animal ecologists to better understand and predict the spatial and temporal patterns of human microbial communities. Explicit theoretical and empirical tests of the hypothesis that principles of community ecology directly apply to human biomedicine also can be found in [44, 52, 53].

*Metacommunity Ecology*. Just as the dispersal of individuals may link the dynamics of subpopulations that are separated in space, dispersal across communities can link local communities into a larger metacommunity. Mittelbach [47] has provided important examples of metacommunity dynamics such as competition-coexistence tradeoffs in patchy environments that we consider to be potentially relevant to human host–human microbiome interactions, and Holt [54] has explored the dynamics of human pathogens in a biogeographical and landscape context.

*Ecosystem Ecology*. The human host can be viewed as an ecosystem composed of interacting populations and communities [3]. Similar to other kinds of ecosystems, the human body exhibits strong and predictable flows of energy and resources, in this case, the inputs and outputs of energy and nutrients that are derived from ingested food and water. Analogous to the differences that exist between pre- and postinvasion nutrient and energy dynamics in pathogen-invaded terrestrial ecosystems [55, 56], pathogen-invaded human bodies can be expected to exhibit changes in nutrient and energy dynamics that vary strongly with the intensity and outcome of infectious disease. Moreover, we suggest that the principles of resource-ratio theory and ecological stoichiometry, which considers how the balance of energy and chemical elements is influenced by organisms and their metabolic activities [57], will apply to the human host and its microbiome. For example, the guts of infants fed breast milk without supplemental iron have been found to develop a microbial community composed primarily of non-toxigenic *Lactobacillus* species; in contrast, infants fed iron-supplemented formula developed a different intestinal community that included potentially toxigenic bacteria such as those belonging to the genera *Clostridium, Salmonella*, and *Staphylococcus* [52].

## THE HUMAN MICROBIOME IN HEALTH AND DISEASE

The human–microbe partnership has coevolved and coadapted into a remarkably stable and diverse biological ecosystem [60, 61], in which the abundance of resident microbial cells exceeds that of our own body cells by approximately 3- to 10-fold [62]. These host-associated microorganisms provide vital ecosystem services that include food processing and digestion [61]; production of vitamins [63]; modulation of epithelial barriers [64]; immune system development and response [65–67]; and defense against invading microbial pathogens [68–70]. The microbiome has been suggested to play a role in modulating behavior, cognition and mood [71, 72].

The community structure and activity of microbial assemblages can vary strongly, both across different sites on an individual body and across different human populations, diets and health conditions [63, 73–77]. While it is widely accepted that infants receive their first major inoculation of microorganisms during the birth process, recent research suggests that initial microbial exposure may occur prior to birth [58, 78, 79]. Both the method of delivery and subsequent environmental exposures (e.g. breastfeeding, skin-to-skin contact between mother and newborn, level of early antibiotic exposure, diseases and childhood diet) reshape the initial microbial community structure, and over time act to establish the microbiota that are characteristic of adults [80–86].

The human microbiome is characterized by a network of microbe–microbe and microbe–host interactions that is typically resistant to modest fluctuations in diet, hormones, immune response and invasions by pathogenic and non-pathogenic microbes [87–89]. Nonetheless, strong perturbations such as treatment with antibiotics can lead to profound changes in microbial community structure from which the microbiome sometimes never completely recovers and can have a substantial influence on human health [90–92]. With the exception of *C. difficile*-associated colitis, the instigating factors that lead to diseases and conditions associated with altered microbial communities are poorly understood. Examples of potential ecological interactions that can occur between the microbial components of the human microbiome and its host are illustrated in Fig. 1.

Culture-independent methods, which permit direct analysis of DNA from a sample rather than cultured microorganisms, enable investigations of many aspects of microbial communities inhabiting the human body. The evolution of knowledge and technology in ecology, microbiology, biochemistry, immunology as well as other fields makes integration of these disciplines with medicine a natural next step. In the four sections later, we have chosen to focus more detailed discussion on the clinical relevance of the gut microbiota, but similar principles can be applied to other human body sites where microbe–microbe and microbe–host interactions can impact health and disease. We also acknowledge that the microbiota of the gut can potentially modulate mental health, immunity and many other clinically relevant issues in human medicine. However, these additional issues are not reviewed here.

## ANTIBIOTICS PERTURB THE GUT MICROBIOME

Historically, medical microbiology has focused primarily on the eradication of pathogens. Common medical practices include the prescription of antibiotics to treat bacterial infections. However, extended use of antibiotics unintentionally perturbs the composition of the human microbiota by killing off indigenous bacteria in addition to pathogens [93]. The removal of indigenous microorganisms invariably affects community composition and the provision of beneficial ecosystem services to humans. Although broad-spectrum antibiotics have saved countless lives and are a necessary tool in fighting off harmful infections, they also select for resistant bacteria [94], increase horizontal gene transfer (which can lead to the spread of antibiotic resistance-associated genes) [95], and are likely to alter microbial physiology and behavior by acting as signaling molecules [96]. During microbial succession following antibiotic treatment, opportunistic pathogens can easily colonize the human body and proliferate without facing intense competition for resources from other members of the local microbial community [97]. Such opportunists include *C. difficile*, which commonly appears in secondary infections in hospital patients previously given antibiotic treatments [98–100]. Antibiotics can significantly influence the composition and function of the human microbiota; the implications of these changes and the ability of the community to resist change or rebound (resilience) are still not fully understood.

Current clinical approaches to ameliorate recurrent *C. difficile* infections include reintroducing microorganisms from a healthy individual to the patient’s perturbed microbiome *via* the provision of probiotics and/or fecal microbiota transplants that amend a *C. difficile*-colonized patient’s gut with microbiota from a non-diseased donor [100]. Both of these therapeutic approaches involve deliberate ecological manipulations of the gut ecosystem. Although the use of probiotics has shown limited success in treating *C. difficile* infections, particularly in patients with recurrent disease [101], fecal microbiota transplants, in contrast, can result in a dramatic decrease in the symptoms of *C. difficile* in patients with chronic recurrent disease and provide strong empirical support for fecal microbiota transplants as a novel therapy [102, 103]. Moreover, fecal microbiota transplants are being considered for approval by the U.S. Food and Drug Administration [102–105], a compelling reason for this ecological approach to be introduced to nascent medical professionals.

## OBESITY, METABOLIC DISORDERS AND INFLAMMATORY DISEASES

Obesity affects 35% of American adults and accounts for 10% of annual U.S. medical expenses [106]. While human genetics, diet and environment all strongly influence the incidence of obesity, a decade of research suggests that the gut microbiome may also play a prominent role [107–109]. For example, gut microbiota transplantation trials in humans found that obese volunteers who received a lean donor microbiota exhibited improved insulin sensitivity over 6-week post-transplant period [110]. In addition, increasing evidence points to the presence of the inflammation-inducing microorganisms in obese individuals, and it has been hypothesized that the development of obesity and metabolic disorders may be linked to chronic gut inflammation [111–113]. Chronic inflammation is at the root of different conditions, including allergies, periodontitis and autoimmune disorders. Inflammation is, therefore, a common problem in almost every branch of medicine, and as such, it is crucial to recognize and understand the ecological processes that can lead to altered microbial community composition and function.

Just as gut microbes have evolved in the nutritional environment of the human intestine, human evolution has been driven by the services that gut microbes provide to health, such as immune system regulation, metabolism and defense against pathogen invasion. Thus, modulations to the gut microbial composition that jeopardize these services could adversely affect human health. Because modifications in diet have been shown to rapidly alter microbial community composition [114], diet manipulation could help treat a number of different metabolic disorders, including inflammatory bowel diseases, obesity and kwashiorkor, a maladaptive phenotype of malnutrition common in the developing world [115]. Standard undergraduate microbiology courses delve into cell physiology and metabolism of microorganisms, topics essential to understanding how our gut microbiota functions and responds to external outputs. Adding instructional material related to microbial community composition and function is an important first step toward understanding how nutrition and supplements could potentially aid in treatment of metabolic disorders.

## POTENTIAL ROLE OF GUT MICROBIOTA IN CANCER DEVELOPMENT

Formal inclusion of the concepts of microbe–microbe and microbe–host interactions during the premedical education, as well as microbial physiology and metabolism, would help to encourage physicians to search for effective methods to impede or prevent microbe-associated cancers. Carcinogens and risk factors for cancer exist within the human microbiome, and cancers induced by microorganisms account for 20% of all fatal cancers in humans [116]. Most known microbially induced cancers are caused by human papilloma viruses, hepatitis B and C viruses and the bacterium *Helicobacter pylori* [117–119]. Recently, Zackular *et al.* [120, 121] have shown that specific microbial communities can drive tumor formation in the colon and that these communities can serve as early biomarkers of tumors.

Cancer is a complex, multifactorial disease and its susceptibility is highly dependent on interactions between human cells and their surrounding environment. Research suggests that microorganisms can potentially modulate tumor growth via three major processes: (i) by metabolizing dietary nutrients into carcinogens or tumor-suppression agents [122, 123]; (ii) by inducing or suppressing inflammation [124–128]; and (iii) by causing DNA damage to host cells [129]. It is worth noting that while many foods have been associated with a heightened risk of cancer, it is the resident microbial community that actively converts them into compounds that can cause DNA damage or inflammation of host tissues [130]. For example, gut microbes produce DNA-damaging compounds *via* fermentation, which may provide the link between the previously established association of red meat consumption and cancer development [122, 130–132]. Likewise, it has been found that commensal *Clostridium* species which, metabolize primary bile acids into carcinogenic deoxycholic acid (DCA), may contribute to the oncogenesis of colorectal and liver cancers [123, 133]. Although antibiotics could theoretically eliminate clostridia that synthesize the unfavorable DCA, tumor suppression was also accomplished in mouse models by inhibiting enzymes in the DCA metabolic pathway [123].

Inflammation triggered by microorganisms is not only a necessary defense mechanism against pathogens but also causes significant damage to host cells and DNA by releasing high amounts of inflammatory signaling molecules and reactive oxygen and nitrogen species [124, 127]. Furthermore, inflammation and cancer are complex processes under the control of many factors, and the underlying mechanisms of their interplay remain obscured. The recruitment of leukocytes, lymphocytes and other inflammatory cells to the site of inflammation results in the release of growth factors and cytokines that could contribute to the progression of tumors by stimulating cell proliferation, differentiation and vascularization [33, 124, 127, 134].

Formal inclusion of the concepts of microbe–microbe and microbe–host interactions, as well as microbial physiology and metabolism during the premedical education would encourage physicians to search for effective methods to impede microbial carcinogen metabolism. For example, negative consequences of commensal microbial metabolism may be better mitigated by targeting specific microbial enzymes and cofactors through selective inhibitors in combination with cancer drugs [135]. It is important to note, however, that the biology behind the microbe–host interaction in the cancer development is complex. For example, while *H. pylori* is a causative agent of gastric cancer, it might have a protective role in esophageal cancer [136, 137].

We stress that our discussion of the human microbiome’s role in cancer has neither touched on the role that commensal microbes play in the metabolism of compounds that reduce the risk of cancers nor explained how commensal microbes might influence inflammation or modify the efficacy of chemotherapeutic agents. However, it does serve to help demonstrate the potential benefits of a paradigm shift in the way premedical and medical students are educated about human-associated microbes and how cancer is studied and treated.

## GUT MICROBIOME EFFECTS ON DRUG PHARMACOKINETICS

The human-associated microbiota also possess diverse metabolic pathways that allow them to directly or indirectly metabolize xenobiotic substrates. Currently, gut microorganisms are known to modulate the metabolism of more than 40 pharmaceutical compounds [138, 139]. For example, *Clostridium sporogenes* plays an important role in reductive metabolism of the anti-seizure drug zonisamide [140], while *Eggerthella lenta* can convert the cardiac glycoside digoxin into an inactive form [138, 141, 142]. Substrate competition between somatic and microbial cells in the gut can also lead to undesirable toxin buildup. For example, anaerobic taxa present in rat feces can convert the analgesic phenacetin into a toxic metabolite associated with methemoglobinemia and nephritis [143]. Furthermore, microbial ß-glucuronidases convert a detoxified species of the cancer drug irinotecan back into its active form, facilitating drug toxicity [144]. Microbial metabolites may also serve as competitive inhibitors to human enzymes; this is the case with the popular over-the-counter analgesic acetaminophen. In individuals whose gut microbiota produce high levels of *p*-cresol, the ability to detoxify acetaminophen is reduced [145]. The microbial metabolite *p*-cresol competitively inhibits a human sulfotransferase, which sulfonates acetaminophen into a non-toxic derivative. The microbial metabolite *p*-cresol may competitively inhibit human sulfotransferases. Unsulfonated or unglucuronidated acetaminophen can be oxidized by the cytochrome P450 system and potentially other oxidative enzymes into hepatotoxic metabolites as reviewed in [146].

It is important to view humans in intimate association with their microbiota, as these microorganisms likely hold the key that explains some of the observed person-to-person variability in drug metabolism and responses. One way to prevent drug toxicity is to screen for the key microorganisms or microbial genes that are involved in drug metabolism. For example, urinalysis can detect production of bacterial metabolites such as *p*-cresol, the metabolite implicated in acetaminophen hepatotoxicity [145]. Community sequencing platforms would be used to screen for the abundance of *Clostridium sporogenes* and *Bifidobacterium bifidum*, the taxa that primarily reduce zonisamide into its inactive form [140]. Matrix-assisted laser desorption/ionization-time of flight (MALDI-TOF) and liquid chromatography/mass spectrometry could detect protein levels of microbial ß-glucuronidases or microbial zonisamide reductases. Another strategy to predict microbial drug transformations would be to administer a tiny dose of the drug and then to analyse patient’s urine or stool samples for microbial derivatives. Physicians could also consider alternate methods of drug administration that result in minimal to no direct contact with the gut microbiome. In one study, inactivated microbial byproducts of digoxin were less abundant in patients with intravenous delivery rather than ingestion [141, 147], perhaps because the intravenous delivery pathway of digoxin largely bypassed the intestinal microbiota.

We suggest that a solid foundation in microbial ecology during premedical education would encourage physicians to be aware of potential microbial influences on drug metabolism and to search for effective means to activate or de-activate microbial metabolisms that interfere with drug effectiveness in their patients. This valuable microbiological perspective on drug administration could assist in the evaluation of drug activity and efficacy and help to prevent or diminish undesirable drug toxicity.

## CONCLUSIONS

Our resident microbiota play important roles in homeostatic physiology and a host of chronic disease conditions. Nearly all of these interactions are mediated through energy and metabolite exchange between human and microbial cells, and through the modulation of human and microbial gene expression [91]. Physicians need access to specific, quantitative metrics that can objectively be used to assess their patients’ health state (e.g. urinalyses; blood cell counts and serum chemistry; concentrations of key enzymes and hormones in tissues and fluids). Physicians also may strongly benefit from analysing their patients’ microbiome for biomarkers that can be used to identify microbe-associated health conditions. For example, we anticipate that microbial methods such as community profiling, metagenomics, RNA sequencing, and MALDI-TOF spectrometry almost certainly will become standard components of diagnostic testing. These important new tools provide doctors with profiles of human microbiome composition and gene expression that can, in turn, be used for disease analysis, tracking and treatment. We strongly believe that an improved knowledge of microbiological concepts will contribute to the improved diagnosis and treatment of human diseases in future.

In this review, we therefore strongly advocate for the inclusion of microbiology and ecology in premedical curricula. As noted by Dienstag [148], ‘A sick patient does not represent a biochemistry problem, an anatomy problem, a genetics problem, or an immunology problem; rather, each person is the product of myriad molecular, cellular, genetic, environmental, and social influences that interact in complex ways to determine health and disease’. Premedical teaching should reflect this diversity and should cut across multiple disciplines.

Although the influences of our microbiota on our health are not yet fully understood, one thing is clear: our bodies cannot be viewed independently of the diverse and highly active microbial inhabitants that interact with us. Because the gut microbiota has global effects on human physiology, the relationship between commensal microbes and the human body is expansive. Although we currently know the most about the gut microbiome and its effects on our health, future studies of the microbiomes of the skin, mouth and other body habitats are equally important. Whether they occur within the gut or elsewhere, the ecological processes that mediate microbial species richness or even the survival of just one microbial taxon, can potentially affect human health.

Finally, we want to state that while work in the human microbiome field is exciting and rapidly progressing, many studies are preliminary and focus on model systems. Further work is required to demonstrate the utility of basic microbiome research to patient populations, another reason that clinicians are such a critical and missing piece of the puzzle.

In our opinion, enhancing undergraduate microbiology and ecology coursework on premedical tracks will provide health professionals with valuable new insights into the human body as a partnership with its microbial inhabitants. With these important foundations in microbial ecology, future medical professionals can pursue clinical training in postgraduate programs with perspectives that will allow them to reassess and reevaluate traditional protocols and treat their patients with foundational microbiome research in mind. Why not start by building a strong understanding and appreciation of the human microbiome in the undergraduate classroom as a standard part of the premedical curricula?
